# Comparison of health-care utilization and expenditures for minimally invasive vs. open colectomy for benign disease

**DOI:** 10.1007/s00464-022-09097-x

**Published:** 2022-02-22

**Authors:** Sarah E. Diaz, Yongjin F. Lee, Amir L. Bastawrous, I.-Fan Shih, Shih-Hao Lee, Yanli Li, Robert K. Cleary

**Affiliations:** 1 Department of Surgery, St Joseph Mercy Hospital Ann Arbor, 5325 Elliott Dr. Suite 104, Ann Arbor, MI 48106 USA; 2 Swedish Cancer Institute, Seattle, WA USA; 3 Global Health Economics and Outcomes Research, Intuitive Surgical, Inc., Sunnyvale, CA USA

**Keywords:** Colon resection, Cost, Health-care utilization, Minimally invasive, Robotic-assisted surgery, Laparoscopic surgery

## Abstract

**Background:**

Adoption of minimally invasive approaches continues to increase, and there is a need to reassess outcomes and cost. We aimed to compare open versus minimally invasive colectomy short- and long-term health-care utilization and payer/patient expenditures for benign disease.

**Methods:**

This is a retrospective analysis of IBM^®^ MarketScan^®^ Database patients who underwent left or right colectomy for benign disease between 2013 and 2018. Outcomes included total health-care expenditures, resource utilization, and direct workdays lost up to 365 days following colectomy. The open surgical approach (OS) was compared to minimally invasive colectomy (MIS) with subgroup analysis of laparoscopic (LS) and robotic (RS) approaches using inverse probability of treatment weighting.

**Results:**

Of 10,439 patients, 2531 (24.3%) had open, 6826 (65.4%) had laparoscopic, and 1082 (10.3%) had robotic colectomy. MIS patients had shorter length of stay (LOS; mean difference, − 1.71, *p* < 0.001) and lower average total expenditures (mean difference, − $2378, *p* < 0.001) compared with open patients during the index hospitalization. At 1 year, MIS patients had lower readmission rates, and fewer mean emergency and outpatient department visits than open patients, translating into additional savings of $5759 and 2.22 fewer days missed from work for health-care visits over the 365-day post-discharge period. Within MIS, RS patients had shorter LOS (mean difference, − 0.60, *p* < 0.001) and lower conversion-to-open rates (odds ratio, 0.31 *p* < 0.001) during the index hospitalization, and lower hospital outpatient visits (mean difference, − 0.31, *p* = 0.001) at 365 days than LS.

**Conclusion:**

MIS colectomy is associated with lower mean health-care expenditures and less resource utilization compared to the open approach for benign disease at index operation and 365-days post-discharge. Health-care expenditures for LS and RS are similar but shorter mean LOS and lower conversion-to-open surgery rates were observed at index operation for the RS approach.

**Supplementary Information:**

The online version contains supplementary material available at 10.1007/s00464-022-09097-x.

The development and introduction of minimally invasive surgery (MIS) tools and approaches have fundamentally changed colorectal surgery. MIS approaches to colorectal surgery offer several outcomes advantages over traditional open surgery (OS) that include earlier return of bowel function, less postoperative pain and opioid use, shorter hospital length of stay (LOS), and fewer surgical site infections [1–5]. The adoption of the MIS approach to colorectal surgery increased from 40 to 60% in 2011 to 75% in 2018 [6]. Early adoption of MIS was initially associated with higher costs due to instrumentation expenses and longer operating times [2]. Changes in reimbursement and bundled payments mandated consideration of short- and long-term costs generated by an index hospital episode [7].

With increasing adoption, it is important to reassess health-care utilization outcomes and cost to determine if the value of MIS has become more favorable with experience. Long-term follow-up potentially reveals robotic surgery (RS) advantages of enhanced vision and articulated instruments that may cause less surgical trauma. This may be associated with decreased health-care expenditures and utilization due to fewer index surgery complications [8]. Previous studies of patients with colorectal cancer have demonstrated the value of MIS, but none have focused on benign disease or assessed value beyond 90 days after the initial hospitalization [8]. The aim of this study is to compare open and minimally invasive colectomy short- and long-term utilization outcomes and payer/patient expenditures for patients with benign disease at the index operation and up to 1 year after surgery with subgroup analysis of laparoscopic (LS) and robotic (RS) approaches.

## Materials and methods

### Data source

This is a retrospective claims data analysis using the IBM® MarketScan® Commercial Claims and Encounter Database (MarketScan®), an aggregated database that contains all paid claims and encounter data generated by approximately 50 million commercially insured individuals in the United States. The database includes inpatient, outpatient, and prescription drug service use, representing the medical experience of insured employees and their dependents [9]. As this was an observational study of de-identified patients in the MarketScan® database, Institutional Review Board approval and consent were exempt (in accordance with the Health Insurance Portability and Accountability Act Privacy Rule).

### Study population

All adults aged 18–64 years in the database with an inpatient colectomy without a colon or rectal cancer diagnosis between January 2013 and December 2018 were identified. We used International Classification of Diseases and Related Health Problems, Ninth and Tenth Revisions, Clinical Modification and Procedure Classification System (ICD-9-CM/ICD-10-CM/ICD-9-PCS/ICD-10-PCS) to define the eligible colectomy cases and differentiate surgical approaches (Supplementary Table 1). To be eligible for data analysis, patients were required to be continuously enrolled with medical and prescription drug coverage from 180 days prior to and 365 days after inpatient colectomy. Exclusion criteria included: (1) emergent cases; (2) inpatient cases that were not coded with diagnosis-related group (DRG) codes 329, 330 or 331; (3) demographic information missing; (4) discharges with extreme total payment in index hospitalization (< 1st or > 99th); (5) patients with capitated payment insurance plans (health maintenance organization and capitated point-of-service) because these plans often submit claims with $0 pay value. Emergent cases were defined as patients who had an emergency room service claim found on the day of admission.

### Outcomes

Study outcomes included mean total health-care expenditures, mean health-care resource utilization and mean direct workdays lost to health-care utilization. These outcomes were assessed during the index surgery and for 365 days post-discharge. The health-care expenditures included both facility and professional payments. Total expenditures were inflation adjusted to 2018 US dollars utilizing the general Consumer Price Index. Health-care utilization included inpatient readmission, emergency department visits, hospital outpatient, and office visits 1-year after the index procedure based on place of service. For direct work loss days due to health-care visits, we converted the LOS to days of utilization for inpatient claims, and assumed a half-day of utilization for an office visit claim, and a full day of utilization for claims related to emergency department, urgent care facility, or other hospital outpatient visit [10, 11].

### Patient factors

Patient-level baseline sociodemographic and clinical characteristics included age, gender, region, insurance plan, metropolitan/non-metropolitan area, the indication for surgery (benign neoplasm, diverticular disease, inflammatory bowel disease), resection type (Left: left hemicolectomy and sigmoidectomy; Right: right hemicolectomy and cecectomy), and year of surgery. Insurance plans were classified into comprehensive insurance, preferred provider organization (PPO), non-capitated point-of-service (POS), and other insurance plans. DRG codes were listed in Table 1 but not included in statistical model. We measured comorbidity using the Charlson Comorbidity Index (CCI) presented at the index hospitalization and in the 180-day preoperative period.

**Table 1:** Baseline demographic characteristics before IPTW adjustment

	Open	MIS	SMD	LAP	RAS	SMD
	(*N* = 2531)	(*N* = 7908)		(*N* = 6826)	(*N* = 1082)	
Age			0.069			0.086
18–44	507 (20.0)	1563 (19.8)		1379 (20.2)	184 (17.0)	
45–54	826 (32.6)	2829 (35.8)		2439 (35.7)	390 (36.0)	
55–64	1198 (47.3)	3516 (44.5)		3008 (44.1)	508 (47.0)	
Sex	1391 (55.0)	4093 (51.8)	0.064	3559 (52.1)	534 (49.4)	0.056
Region			0.110			0.077
North Central	641 (25.3)	1839 (23.3)		1560 (22.9)	279 (25.8)	
Northeast	347 (13.7)	1287 (16.3)		1127 (16.5)	160 (14.8)	
South	1284 (50.7)	3795 (48.0)		3289 (48.2)	506 (46.8)	
West	259 (10.2)	987 (12.5)		850 (12.5)	137 (12.7)	
Insurance plan			0.078			0.063
PPO	1680 (66.4)	5303 (67.1)		4593 (67.3)	710 (65.6)	
Comprehensive	151 (6.0)	354 (4.5)		294 (4.3)	60 (5.5)	
Non-capitated POS	214 (8.5)	614 (7.8)		533 (7.8)	81 (7.5)	
Others	486 (19.2)	1637 (20.7)		1406 (20.6)	231 (21.3)	
Charlson comorbidity			0.165			0.053
0	1387 (54.8)	4836 (61.2)		4192 (61.4)	644 (59.5)	
1	556 (22.0)	1738 (22.0)		1501 (22.0)	237 (21.9)	
≥ 2	588 (23.2)	1334 (16.9)		1133 (16.6)	201 (18.6)	
Metropolitan	519 (20.5)	1047 (13.2)	0.195	905 (13.3)	142 (13.1)	0.004
DRG			0.433			0.095
329	516 (20.4)	801 (10.1)		702 (10.3)	99 (9.1)	
330	1314 (51.9)	3432 (43.4)		2996 (43.9)	436 (40.3)	
331	701 (27.7)	3675 (46.5)		3128 (45.8)	547 (50.6)	
Inflammatory bowel disease	451 (17.8)	865 (10.9)	0.197	809 (11.9)	56 (5.2)	0.241
Benign colon neoplasm	552 (21.8)	2352 (29.7)	0.182	2023 (29.6)	329 (30.4)	0.017
Diverticular disease	1463 (57.8)	5017 (63.4)	0.116	4286 (62.8)	731 (67.6)	0.100
Year			0.090			0.488
2013	504 (19.9)	1529 (19.3)		1419 (20.8)	110 (10.2)	
2014	624 (24.7)	1875 (23.7)		1703 (24.9)	172 (15.9)	
2015	569 (22.5)	1735 (21.9)		1515 (22.2)	220 (20.3)	
2016	472 (18.6)	1381 (17.5)		1108 (16.2)	273 (25.2)	
2017	362 (14.3)	1388 (17.6)		1081 (15.8)	307 (28.4)	
Baseline total expenditures						
Mean ± SD	$24297 ± $33552	$17805 ± $23445	0.224	$17988 ± $23866	16652 ± 20567	0.060
Median (Q1, Q3)	$13579 ($5941, $28625)	$10678 ($5175, $21479)		$10758 ($5227, $21736)	$10266 ($4712, $19728)	

*IPTW* inverse probability of treatment weighting, *SMD* standard mean difference, *MIS* minimally invasive surgery, *LS* laparoscopic surgery, *RS* robotic surgery, *POS* Point-of-Service, *PPO* preferred provider organization, *DRG* Diagnosis-Related Group, *DRG 329/330/331* major small and large bowel procedures

### Statistical analysis

All descriptive and statistical testing analyses were conducted comparing the open surgical approach to minimally invasive surgical approaches, and between LS and RS. Patient characteristics at baseline were summarized as frequencies with proportions for categorical variables and means with standard deviation for continuous variables. Inverse probability of treatment weighting (IPTW) was conducted to minimize the effect of potential confounding factors without reducing the sample size [12]. We applied stabilized propensity score weights to estimate average treatment effect using a logistic regression model with all the baseline patient factors mentioned above. The covariates were selected based on prior knowledge and literature [10, 11, 13]. After IPTW, covariates were considered balanced if the absolute value of the standardized mean difference (SMD) was less than 0.10. Generalized linear model and logistic regression, weighted by the IPTWs and adjusting for the total health-care expenditures in the 180-day preoperative period (i.e., baseline expenditures) were used to estimate the health-care expenditures and utilization. Expenditures, hospital outpatient and office utilization and estimated days off from work were estimated using gamma distribution; emergent department and inpatient services were modeled using zero-inflated Poisson in the generalized linear models. All analyses were performed using SAS software version 9.4 (SAS Institute Inc., Cary, NC) and a 2-tailed *p* < 0.05 was considered statistically significant.

## Results

**Fig. 1:**
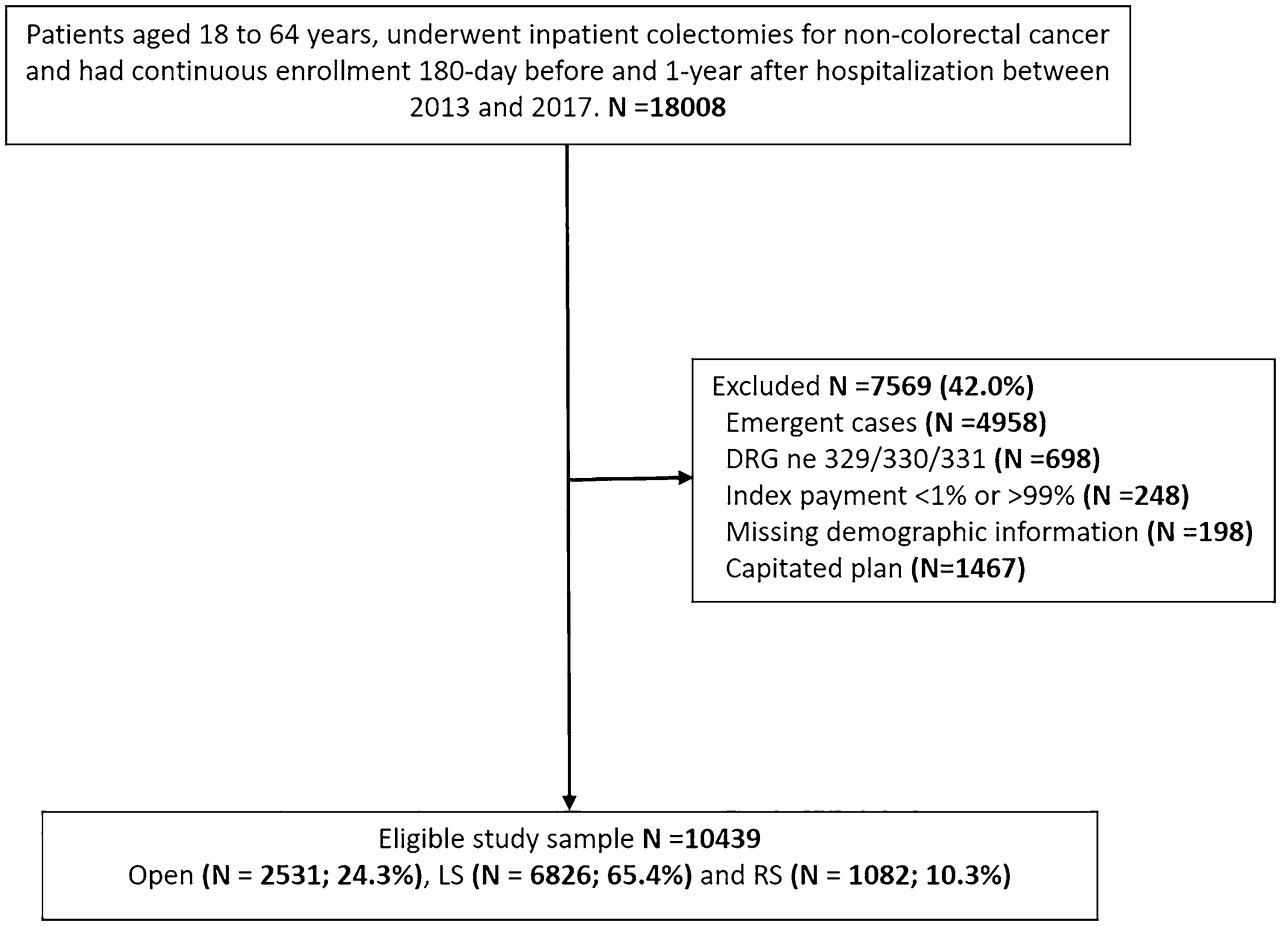
Study Flow. *DRG* Diagnosis-Related Group, *LS* laparoscopic surgery, *RS* robotic surgery

After excluding emergent cases (*n* = 4958), cases without DRG 329/330/331 codes (*n* = 698), index payments < 1st or > 99th (*n* = 248), cases with missing demographic information (*n* = 198), and cases with capitated plans (*n* = 1467), there were 10,439 eligible patients in the dataset, including 2531 (24.3%) OS, 6826 (65.4%) LS, and 1082 (10.3%) RS (Fig. 1). Table 1 shows baseline sociodemographic characteristics before IPTW. There were several differences in SMD before IPTW that included Region (SMD = 0.110) and DRG codes (SMD = 0.433). The OS group had significantly more patients with two or more comorbidities (23.2% vs 16.9%, SMD = 0.165), more patients located in metropolitan area (20.5% vs 13.2%, SMD = 0.195), more with inflammatory bowel disease (17.8% vs 10.9%, SMD = 0.197), and higher total baseline health-care expenditures (mean 24,297 vs $17,805, SMD = 0.224). The OS group had significantly fewer patients with benign neoplasm (21.8% vs 29.7%, SMD = 0.182) and diverticular disease (57.8% vs 63.4%, SMD = 0.116). The only significant difference between LS and RS groups prior to IPTW was the number of patients with diverticular disease (LS 62.8% vs RS 67.6%, SMD = 0.100). After IPTW, there were no significant sociodemographic differences between OS and MIS or between LS and RS groups (Supplementary Table 2).

**Fig. 2:**
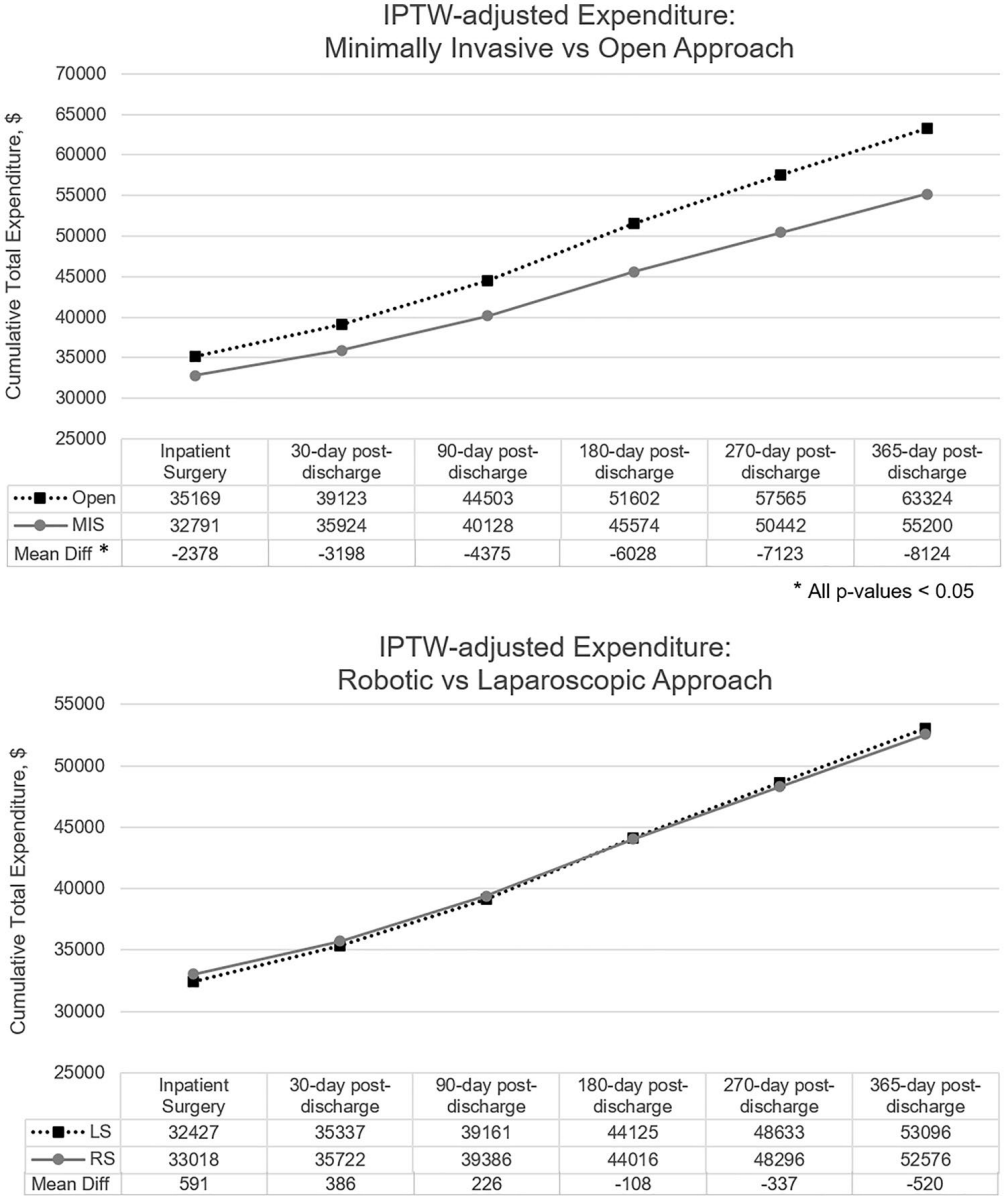
Time series graphics for the IPTW-adjusted expenditures. Total health-care expenditure was calculated by adding hospital and physician payments during the inpatient stay (index surgery) and all health services related costs within the 1-year after discharge, including inpatient, outpatient, and prescription drug services cumulatively. *IPTW* inverse probability of treatment weighting *MIS* minimally invasive surgery, *LS* laparoscopic surgery, *RS* robotic surgery *p < 0.05

Figure 2 shows IPTW-adjusted mean total expenditures between groups starting with the index surgery hospital stay, and up to 365 days post-discharge. Mean total expenditures were significantly higher for OS colectomy than for MIS colectomy at all time periods analyzed—index surgery episode ($35,169 vs $32,791, *p* < 0.001), from index to 30-day post-discharge ($39,123 vs $35,924, *p* < 0.001), 90-day post-discharge ($44,503 vs $40,128, *p* < 0.001), 180-day post-discharge ($51,602 vs $45,574, *p* < 0.001), 270-day post-discharge ($57,565 vs $50,442, *p* < 0.001), and 365-day post-discharge ($63,324 vs $55,200, *p* < 0.001). Subgroup analysis of LS vs RS revealed no significant differences in mean payer expenses for all time periods analyzed.

For IPTW-adjusted resource utilization, MIS showed several favorable outcomes when compared to the OS approach (Table 2). At the index surgery, LOS (mean 5.91 vs 4.20 days, *p* < 0.001) and mean hospital payments ($30,176 vs $27,746, *p* < 0.001) were significantly more for OS colectomy than for MIS. At 365 days after discharge, MIS patients were less likely to be readmitted (OR 0.53, *p* < 0.001), visit the emergency department (OR 0.88, *p* = 0.008) and visit the hospital outpatient department (OR 0.74, *p* < 0.001) than OS patients. MIS patients also had lower mean inpatient LOS (mean difference, − 0.99 days, *p* < 0.001), lower number of emergency department visits (mean difference, − 0.08, *p* < 0.001), and lower number of hospital outpatient visits (mean difference, − 1.10, *p* < 0.001). The reduction in health-care use among MIS patients translated into additional savings of $5759 and 2.22 days (both *p* < 0.001) fewer days missed from work due to health-care visits over the 1-year post-discharge period.

**Table 2:** IPTW-adjusted differences in health-care utilization and expenditures between open and MIS colectomies

	Open		MIS		Adjusted differences (MIS—Open)		*p* Value
	Mean	%	Mean	%	Mean (95% CI)	OR (95% CI)	
Index surgery							
LOS, days	5.91	NA	4.20	NA	− 1.71 (− 1.84, − 1.58)	NA	< 0.001
Hospital payment, dollars	30176	NA	27746	NA	− 2430 (− 3097, − 1763)	NA	< 0.001
Physician payment, dollars	2794	NA	2866	NA	72 (− 54, 198)	NA	0.263
Total payment, dollars	35169	NA	32791	NA	− 2378 (− 3082, − 1673)	NA	< 0.001
1-year post surgery							
Total payment, dollars	28442	NA	22684	NA	− 5759 (− 7438, − 4079)	NA	< 0.001
Readmission, %	NA	28.4	NA	17.3	NA	0.53 (0.48, 0.59)	< 0.001
Inpatient LOS	2.21	NA	1.21	NA	− 0.99 (− 1.15, − 0.83)	NA	< 0.001
ER visit, %	NA	34.6	NA	31.7	NA	0.88 (0.80, 0.97)	0.008
Number of ER visits	0.66	NA	0.58	NA	− 0.08 (− 3.35, − 0.13)	NA	< 0.001
Hospital Outpatient visit, %	NA	79.7	NA	74.4	NA	0.74 (0.67, 0.83)	< 0.001
Number of outpatient visits	5.15	NA	4.05	NA	− 1.10 (− 1.25, − 0.95)	NA	< 0.001
Number of office visits	11.50	NA	11.43	NA	− 0.07 (− 0.26, 0.12)	NA	0.448
Number of days off	14.09	NA	11.87	NA	− 2.22 (− 2.79, − 1.65)	NA	< 0.001

*IPTW* inverse probability of treatment weighting, *MIS* minimally invasive surgery, *CI* confidence interval, *OR* odds ratio, *LOS* length of stay, *ER* emergent room

Subgroup analysis of LS vs RS shows that index LOS (Table 3; mean 4.22 vs 3.63 days, *p* < 0.001), conversion-to-open (8.0% vs 2.6%, *p* < 0.001), and mean payments to physicians ($2872 vs $2653, *p* = 0.011) were significantly more for LS than for RS. In addition, the average number of outpatient hospital visits at 1 year was significantly higher for the LS approach (mean difference, − 0.31, *p* = 0.001). There were no significant differences between LS and RS for all other outcomes and payments.

**Table 3:** IPTW-adjusted differences in health-care utilization and expenditures between laparoscopic and robotic colectomies

	LS		RS		Adjusted differences (RS—LS)		*p* Value
	Mean	%	Mean	%	Mean (95% CI)	OR (95% CI)	
Index surgery							
LOS, days	4.22	NA	3.63	NA	− 0.60 (− 0.72, − 0.47)	NA	< 0.001
Conversion, %	NA	8.0	NA	2.6	NA	0.31 (0.21, 0.46)	< 0.001
Hospital payment, dollars	27421	NA	27937	NA	516 (− 368, 1400)	NA	0.252
Physician payment, dollars	2872	NA	2653	NA	− 219 (− 386, − 51)	NA	0.011
Total payment, dollars	32427	NA	33018	NA	591 (− 344, 1525)	NA	0.190
1-year post surgery							
Total payment, dollars	20929	NA	19872	NA	− 1057 (− 2829, 715)	NA	0.070
Readmission, %	NA	16.7	NA	16.4	NA	0.95 (0.82, 1.09)	0.441
Inpatient LOS	1.13	NA	1.31	NA	0.18 (− 0.02, 0.38)	NA	0.170
ER visit, %	NA	31.5	NA	31.6	NA	1.00 (0.87, 1.15)	0.994
Number of ER visits	0.58	NA	0.56	NA	− 0.01 (− 0.07, 0.05)	NA	0.732
Hospital Outpatient visit, %	NA	73.7	NA	72.6	NA	0.98 (0.83, 1.16)	0.819
Number of outpatient visits	3.84	NA	3.54	NA	− 0.31 (− 0.49, − 0.12)	NA	0.001
Number of office visits	11.25	NA	11.18	NA	− 0.07 (− 0.33, 0.19)	NA	0.614
Number of days off	11.47	NA	11.33	NA	− 0.14 (− 0.83, 0.54)	NA	0.680

*IPTW* inverse probability of treatment weighting, *LS* laparoscopic surgery, *RS* robotic surgery, *CI* confidence interval, *OR* odds ratio, *LOS* length of stay, *ER* emergent room

The impact of MIS conversion-to-open is shown in Table 4. At the index hospitalization, LOS (mean 5.44 vs 4.04 days, *p* < 0.001) and hospital payments (mean $30,012 vs $27,263, *p* < 0.001) were significantly more for MIS cases requiring conversion. At 1 year, converted cases had significantly more readmissions (23.2% vs 16.0%, *p* < 0.001), inpatient hospital days (mean difference, − 1.13 days, *p* < 0.001), outpatient hospital visits (79.4% vs 73.2%, *p* = 0.002) and average number of outpatient hospital visits (mean difference, -0.41, *p* < 0.002). This translated into additional savings of $4816 and 1.46 fewer days missed from work for health-care visits over the 1-year post-discharge period.

**Table 4:** IPTW-adjusted differences in health care utilization and expenditures between MIS-complete and converted colectomies

	MIS-Complete		Converted		Adjusted differences (MIS—Open)		*p* Value
	Mean	%	Mean	%	Mean (95% CI)	OR (95% CI)	
Index surgery							
LOS, days	4.04	NA	5.44	NA	1.39 (1.16, 1.63)	NA	< 0.001
Hospital payment, dollars	27263	NA	30012	NA	2749 (1508, 3990)	NA	< 0.001
Physician payment, dollars	2850	NA	2778	NA	− 71 (− 301, 158)	NA	0.542
Total payment, dollars	32286	NA	35028	NA	2742 (1445, 4039)	NA	< 0.001
1-year post surgery							
Total payment, dollars	20332	NA	25148	NA	4816 (1938, 7693)	NA	0.001
Readmission, %	NA	16.0	NA	23.2	NA	1.59 (1.29, 1.96)	< 0.001
Inpatient LOS	1.03	NA	2.16	NA	− 1.13 (− 0.77, − 1.49)	NA	< 0.001
ER visit, %	NA	31.3	NA	31.4	NA	1.00 (0.83, 1.21)	0.974
Number of ER visits	0.57	NA	0.58	NA	− 0.02 (0.07, − 0.11)	NA	0.696
Hospital Outpatient visit, %	NA	73.2	NA	79.4	NA	1.41 (1.14, 1.75)	0.002
Number of outpatient visits	3.80	NA	4.21	NA	− 0.41 (− 0.16, − 0.67)	NA	0.002
Number of office visits	11.26	NA	10.88	NA	0.39 (0.76, 0.01)	NA	0.045
Number of days off	11.32	NA	12.78	NA	1.46 (0.46, 2.46)	NA	0.004

*IPTW* inverse probability of treatment weighting, *MIS* minimally invasive surgery, *CI* confidence interval, *OR* odds ratio, *LOS* length of stay, *ER* emergent room

Diverticular disease was the most common benign disease diagnosis, comprising 48.2% of the OS group and 55.4% of the MIS group (Supplementary Table 3 and Supplementary Figure S1). Most of the health-care utilization and expenditure outcomes for this subgroup were similar to the overall study results. Exceptions were that there was a significant difference in index surgery physician payments (mean OS $2613 vs MIS $2887, *p* = 0.015) and no significant difference in ER visits (OS 33.9% vs 33.6%, *p* = 0.848) for patients with diverticular disease.

## Discussion

Previous studies have been limited by grouping together benign and malignant diagnoses, colon and rectal disease, or by limiting the analysis to malignant disease [7, 8, 14–17]. This large, national claims data analysis focuses on colectomy for benign disease and shows that MIS colectomy for benign disease is associated with lower mean health-care utilization and payer/patient expenses than open colectomy in both short- and long-term postoperative periods. Specifically, MIS is associated with shorter mean hospital LOS at the index hospitalization as well as lower readmission rates, mean number of ED and hospital outpatient department visits, and mean number of days missed from work during the first year after surgery. In addition, RS patients had shorter mean index hospital LOS, lower conversion-to-open surgery rates and less mean hospital outpatient visits after surgery when compared to the LS group. These findings likely reflect long-term MIS colectomy benefits that include faster recovery with fewer complications and less pain [1–5].

Previous studies have shown mixed results for the cost advantage of MIS and for what category advantages are most apparent [7, 8, 18, 19]. A large, regional risk-adjusted database study composed of Blue Cross Blue Shield of Michigan and Medicare price-standardized payments showed that costs associated with MIS colorectal surgery were significantly less than OS, even after accounting for the cost of conversion. The cost advantage for MIS was most evident in the index hospitalization and post-discharge care up to 90 days [7]. A population-based study of Medicare beneficiaries showed that the LS cost savings when compared to open colectomy were due to lower expenditures for complications, readmissions, and post-acute care [18]. Another New York State Cancer Registry analysis showed that that there was no 90-day cost benefit for MIS over OS but that 90-day utilization, represented by hospital days, was decreased for both LS and RS approaches when compared to OS [8]. In contrast, our study showed significantly decreased expenditures associated with the MIS approach for all index hospitalization categories except physician payments and for post-discharge expenditures at all time periods up to one year after surgery. For health-care utilization, our study is consistent with a previous study showing decreased LOS and readmissions [8]. Our study also showed decreased mean number of ED and outpatient department visits for MIS compared to OS.

The cost of laparoscopic instrumentation, operative times, and concerns about the possible negative impact on outcomes limited wide implementation of LS in colorectal surgery following the first report in 1991 [20]. For those mastering the learning curve and with LS experience, cost and outcomes studies ultimately demonstrated the benefit of this approach [7, 21]. Similarly, institutional costs of RS precluded wider implementation in some hospitals [7, 14], and the value of RS, when considering outcomes and cost, is currently debated in the literature [22, 23]. Like laparoscopic colorectal surgery, the cost of RS has decreased with time and experience, likely due to reductions in operative time, LOS, conversion-to-open, standardized surgical protocols, and surgeon volume defined as ≥ 30 cases per year [8, 24–26]. Previous studies have shown mixed results comparing LS and RS cost-effectiveness [2, 8, 16, 24, 27]. A National Inpatient Sample database comparison of LS and RS sigmoid colectomy showed that LOS was significantly shorter for RS but that total hospital charges were significantly higher [2]. In contrast to our study, this database analysis relied on ICD-9 procedure codes and hospital charges, included an earlier time period than ours (2014 *vs* 2018), and may have included more patients in the robotic learning curve with longer operative times and more instrument needs.

Using an incremental cost-effectiveness ratio, estimated using overall costs and quality-adjusted life years, another recent comparison of LS and RS approaches for right colon cancer showed that there was no significant difference in costs between groups and that there was a 78.78–95.04% probability that the RS group was more cost effective compared to the LS group [27]. Our study showed no difference in mean expenditures between RS and LS approaches for all categories except physician payments (LS > RS, *p* = 0.01) at index surgery. In a subgroup analysis, we observed higher mean expenditures and utilization for converted than for non-converted MIS cases at index surgery and 1-year after surgery.

This study is retrospective with inherent limitations. These include associated biases (surgeon being the most glaring). The database also depends on dependable data entry, and ICD-9 and ICD-10 coding accuracy for surgical procedures. Patients were assigned the robotic approach if robotic and laparoscopic or open codes were identified in the same index surgery claim. The database lacks granular detail for surgical procedures and may potentially have absent data. There is the possibility of unidentified confounders due to unmeasured characteristics. The IBM^®^MarketScan^®^ database does not account for surgeon selection bias choosing the operative approach, variations in surgeon volume, and enhanced recovery care elements that may impact outcomes and expenditures. This is a study of patients with colectomies and benign disease. The results may not be generalizable to those uninsured or having rectal resections. We are currently evaluating malignant disease in a separate analysis. The strengths of this study are the ability to evaluate real-world claims data rather than direct costs or charges associated with in-hospital care or the operative intervention, and using population-based data that represents surgeons and hospitals of varying degrees of expertise. Furthermore, the ability to evaluate the impact on outpatient expenditures and resource utilization up to one year after surgery are particular advantages to this study. This study adds to the cost analysis of colorectal surgery from a unique perspective.

These data suggest that the continued increase in MIS adoption may translate into continued cost savings. Future cost analyses will likely include short-term considerations such as the cost of conversion and long-term considerations such as the ability to perform intracorporeal anastomoses that allow off-midline specimen extraction sites, thereby decreasing hernia rates with the associated morbidity and cost [7, 28, 29].

### Conclusion

Minimally invasive colectomy is associated with lower mean health-care expenditures and less mean health-care resource utilization compared to the open approach for benign disease at the time of index surgery and at 365-days post-discharge. Health-care expenditures for laparoscopic and robotic colectomy are not significantly different, but shorter mean LOS and lower conversion-to-open surgery rates were observed at index operation for the RS approach. Future studies should consider other operative approach procedural differences that may impact health-care expenditures and resource utilization.

## Supplementary Information

Below is the link to the electronic supplementary material.

Supplementary file1 (TIF 145 KB) Supplemental Fig. S1 Time series graphics for the IPTW-adjusted expenditures among patients with diverticular disease only. Cumulative total health-care expenditure was calculated by adding hospital and physician payments during the inpatient stay (index surgery) and all health services related costs within the 1-year after discharge, including inpatient, outpatient, and prescription drug services cumulatively. *IPTW* inverse probability of treatment weighting, *MIS* minimally invasive surgery, *mean diff* mean difference *p<0.05

Supplementary file2 (DOCX 14 KB)

Supplementary file3 (DOCX 19 KB)

Supplementary file4 (DOCX 15 KB)
