# Supplementary figures and images for: Comparison of health-care utilization and expenditures for minimally invasive vs. open colectomy for benign disease
Source: Surg Endosc. 2022 Feb 22;36(10):7250–8. doi: 10.1007/s00464-022-09097-x (PMC9485164; doi:10.1007/s00464-022-09097-x)

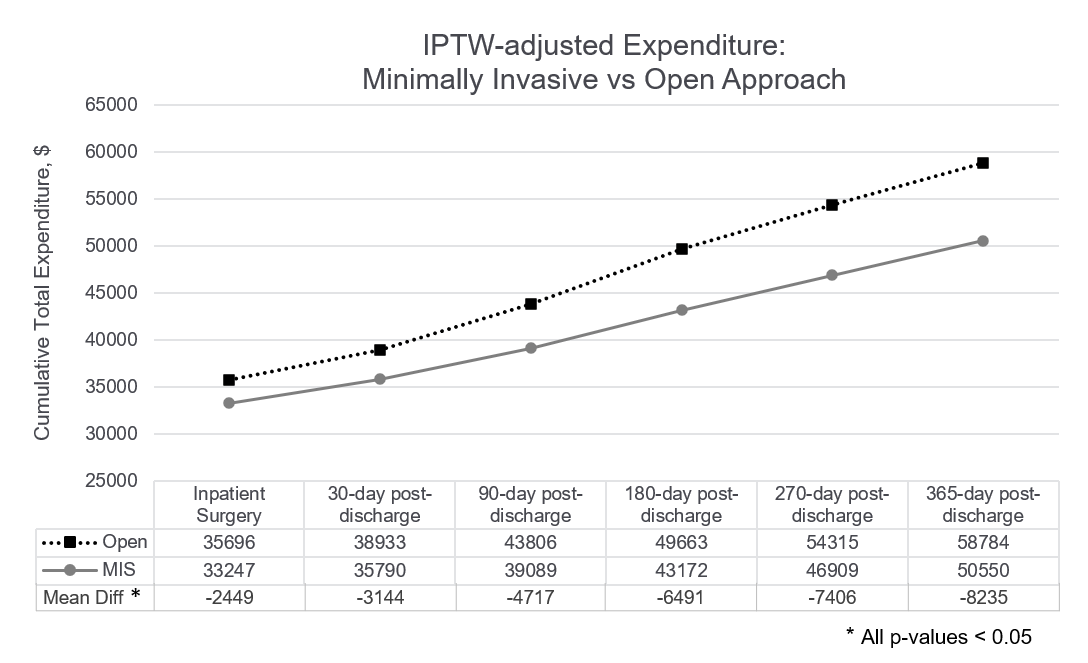

Supplement: Supplementary file 1 — Supplementary file1 (TIF 145 KB) Supplemental Fig. S1 Time series graphics for the IPTW-adjusted expenditures among patients with diverticular disease only. Cumulative total health-care expenditure was calculated by adding hospital and physician payments during the inpatient stay (index surgery) and all health services related costs within the 1-year after discharge, including inpatient, outpatient, and prescription drug services cumulatively. IPTW inverse probability of treatment weighting, MIS minimally invasive surgery, mean diff mean difference *p<0.05 [file 464_2022_9097_MOESM1_ESM.tif]
