# Supplementary material for: Comparison of health-care utilization and expenditures for minimally invasive vs. open colectomy for benign disease
Source: Surg Endosc. 2022 Feb 22;36(10):7250–8. doi: 10.1007/s00464-022-09097-x (PMC9485164; doi:10.1007/s00464-022-09097-x)
Supplement: Supplementary file 2 — Supplementary file2 (DOCX 14 KB) [file 464_2022_9097_MOESM2_ESM.docx]

**Supplementary Table 1: Procedure codes used for eligibility**

| **Measure** | **Grouping** | **Code type** | **Code** |
| --- | --- | --- | --- |
| Left colectomy | Laparoscopic | ICD-9- PCS | 17.35, 17.36 |
|  |  | ICD-10-PCS | 0DTM4ZZ, 0DTG4ZZ, 0DTN4ZZ |
|  | Open | ICD-9- PCS | 45.75, 45.76 |
|  |  | ICD-10-PCS | 0DTM0ZZ, 0DTG0ZZ, 0DTN0ZZ |
| Right colectomy | Laparoscopic | ICD-9- PCS | 17.32, 17.33 |
|  |  | ICD-10-PCS | 0DTF4ZZ, 0DTH4ZZ, 0DTK4ZZ |
|  | Open | ICD-9- PCS | 45.72, 45.73 |
|  |  | ICD-10-PCS | 0DTF0ZZ, 0DTH0ZZ,0DTK0ZZ |
| Robotic surgery | N/A | CPT | S2900 |
|  |  | ICD-9- PCS | 17.4x |
|  |  | ICD-10-PCS | 8E0**CZ |
| Conversion | Conversion | ICD-9- CM | V64.41 |
|  |  | ICD-10-CM | Z53.31, Z53.39 |
|  | Conversion via inspection | ICD-10-PCS | 0DJD4ZZ |

ICD-9-PCS/ICD-10-PCS, International Classification of Diseases, 9th and 10th Procedure Classification System; CPT, Current Procedural Terminology; ICD-9-CM/ICD-10-CM, International Classification of Diseases, 9th and 10th Clinical Modification.
