# Supplementary material for: Comparison of health-care utilization and expenditures for minimally invasive vs. open colectomy for benign disease
Source: Surg Endosc. 2022 Feb 22;36(10):7250–8. doi: 10.1007/s00464-022-09097-x (PMC9485164; doi:10.1007/s00464-022-09097-x)
Supplement: Supplementary file 3 — Supplementary file3 (DOCX 19 KB) [file 464_2022_9097_MOESM3_ESM.docx]

**Supplementary Table 2. Baseline demographic characteristics after inverse probability of treatment weighting (IPTW) adjustment.**

|  | Open | MIS | SMD |  | LS | RS | SMD |
| --- | --- | --- | --- | --- | --- | --- | --- |
|  | (N=2539) | (N=7913) |  |  | (N=6825) | (N=1090) |  |
| Age |  |  | 0.005 |  |  |  | 0.019 |
| 18-44 | 506.7 (20.0) | 1575.1 (19.9) |  |  | 1349.3 (19.8) | 223.4 (20.5) |  |
| 45-54 | 894.0 (35.2) | 2770.7 (35.0) |  |  | 2440.9 (35.8) | 383.9 (35.2) |  |
| 55-64 | 1138.0 (44.8) | 3567.2 (45.1) |  |  | 3034.8 (44.5) | 482.7 (44.3) |  |
| Sex | 1316.2 (51.8) | 4148.2 (52.4) | 0.011 |  | 3533.6 (51.8) | 576.6 (52.9) | 0.023 |
| Region |  |  | 0.009 |  |  |  | 0.069 |
| North Central | 609.8 (24.0) | 1878.2 (23.7) |  |  | 1585.9 (23.2) | 240.6 (22.1) |  |
| Northeast | 394.9 (15.6) | 1236.4 (15.6) |  |  | 1111.8 (16.3) | 184.3 (16.9) |  |
| South | 1236.2 (48.7) | 3852.3 (48.7) |  |  | 3278.6 (48.0) | 549.1 (50.4) |  |
| West | 297.8 (11.7) | 946.1 (12.0) |  |  | 848.8 (12.4) | 116.1 (10.7) |  |
| Insurance Plan |  |  | 0.008 |  |  |  | 0.021 |
| PPO | 1704.5 (67.1) | 5297.5 (66.9) |  |  | 4577.8 (67.1) | 730.6 (67.0) |  |
| Comprehensive | 126.8 ( 5.0) | 386.6 ( 4.9) |  |  | 304.9 ( 4.5) | 46.2 ( 4.2) |  |
| Non-capitated POS | 197.3 ( 7.8) | 623.2 ( 7.9) |  |  | 530.3 ( 7.8) | 90.1 ( 8.3) |  |
| Others | 510.0 (20.1) | 1605.7 (20.3) |  |  | 1412.1 (20.7) | 223.2 (20.5) |  |
| Charlson Comorbidity |  |  | 0.005 |  |  |  | 0.020 |
| 0 | 1517.0 (59.8) | 4715.9 (59.6) |  |  | 4173.2 (61.1) | 675.5 (62.0) |  |
| 1 | 558.7 (22.0) | 1738.8 (22.0) |  |  | 1500.7 (22.0) | 238.0 (21.8) |  |
| >=2 | 463.0 (18.2) | 1458.3 (18.4) |  |  | 1151.1 (16.9) | 176.5 (16.2) |  |
| Metropolitan | 386.6 (15.2) | 1188.0 (15.0) | 0.006 |  | 904.7 (13.3) | 154.2 (14.1) | 0.026 |
| DRG |  |  | 0.335 |  |  |  | 0.073 |
| 329 | 480.6 (18.9) | 835.4 (10.6) |  |  | 700.6 (10.3) | 101.7 ( 9.3) |  |
| 330 | 1275.7 (50.2) | 3501.9 (44.3) |  |  | 2983.5 (43.7) | 447.1 (41.0) |  |
| 331 | 782.4 (30.8) | 3575.7 (45.2) |  |  | 3140.9 (46.0) | 541.3 (49.7) |  |
| Inflammatory bowel disease | 329.7 (13.0) | 1011.0 (12.8) | 0.006 |  | 746.8 (10.9) | 125.1 (11.5) | 0.017 |
| Benign colon neoplasm | 738.4 (29.1) | 2204.6 (27.9) | 0.027 |  | 2031.2 (29.8) | 331.6 (30.4) | 0.014 |
| Diverticular disease | 1597.9 (62.9) | 4910.8 (62.1) | 0.018 |  | 4329.8 (63.4) | 695.9 (63.8) | 0.008 |
| Year |  |  | 0.011 |  |  |  | 0.017 |
| 2013 | 489.0 (19.3) | 1538.8 (19.4) |  |  | 1320.0 (19.3) | 214.4 (19.7) |  |
| 2014 | 607.7 (23.9) | 1891.8 (23.9) |  |  | 1618.6 (23.7) | 264.1 (24.2) |  |
| 2015 | 552.3 (21.8) | 1745.1 (22.1) |  |  | 1497.7 (21.9) | 236.5 (21.7) |  |
| 2016 | 457.0 (18.0) | 1407.2 (17.8) |  |  | 1191.6 (17.5) | 187.7 (17.2) |  |
| 2017 | 432.5 (17.0) | 1330.1 (16.8) |  |  | 1197.1 (17.5) | 187.3 (17.2) |  |
| Baseline total payment |  |  |  |  |  |  |  |
| mean ± SD | 19696 ± 26051 | 19786 ± 29853 | 0.003 |  | 17800 ± 23442 | 17214 ± 21467 | 0.026 |

IPTW, inverse probability of treatment weighting; SMD, standard mean difference; MIS, minimally invasive surgery; LS, laparoscopic surgery; RS, robotic surgery; POS, Point-of-Service; PPO, preferred provider organization; DRG, Diagnosis Related Group; DRG 329/330/331, major small and large bowel procedures.
