# Supplementary material for: Comparison of health-care utilization and expenditures for minimally invasive vs. open colectomy for benign disease
Source: Surg Endosc. 2022 Feb 22;36(10):7250–8. doi: 10.1007/s00464-022-09097-x (PMC9485164; doi:10.1007/s00464-022-09097-x)
Supplement: Supplementary file 4 — Supplementary file4 (DOCX 15 KB) [file 464_2022_9097_MOESM4_ESM.docx]

**Supplementary Table 3. IPTW-adjusted differences in health care utilization and expenditures between open and MIS colectomies among patients with diverticular disease only**

|  | Open | |  | MIS | |  | Adjusted differences (MIS - Open) | |  |
| --- | --- | --- | --- | --- | --- | --- | --- | --- | --- |
|  | Mean | % |  | Mean | % |  | Mean (95% CI) | OR (95% CI) | P-Value |
| Index surgery |  |  |  |  |  |  |  |  |  |
| LOS, days | 5.97 | NA |  | 4.15 | NA |  | -1.83 (-2.00, -1.65) | NA | <.001 |
| Hospital payment, dollars | 30690 | NA |  | 28019 | NA |  | -2672 (-3628, -1715) | NA | <.001 |
| Physician payment, dollars | 2613 | NA |  | 2887 | NA |  | 213 (42, 384) | NA | 0.015 |
| Total payment, dollars | 35696 | NA |  | 33247 | NA |  | -2449 (-3446, -1452) | NA | <.001 |
| 1-year post surgery |  |  |  |  |  |  |  |  |  |
| Total payment, dollars | 23345 | NA |  | 17567 | NA |  | -5778 (-7704, -3853) | NA | 0.001 |
| Readmission, % | NA | 30.6 |  | NA | 16.7 |  | NA | 0.46 (0.39, 0.53) | <.001 |
| Inpatient LOS | 2.22 | NA |  | 1.12 | NA |  | -1.10 (-1.32, -0.88) | NA | <.001 |
| ER visit, % | NA | 33.9 |  | NA | 33.6 |  | NA | 0.99 (0.86, 1.13) | 0.848 |
| Number of ER visits | 0.64 | NA |  | 0.63 | NA |  | -0.01 (-0.08, 0.06) | NA | 0.762 |
| Hospital Outpatient visit, % | NA | 77.8 |  | NA | 71.4 |  | NA | 0.71 (0.61, 0.83) | <.001 |
| Number of outpatient visits | 4.25 | NA |  | 3.50 | NA |  | -0.75 (-0.95, -0.56) | NA | <.001 |
| Number of office visits | 11.04 | NA |  | 11.29 | NA |  | 0.25 (-0.02, 0.52) | NA | 0.073 |
| Number of days off | 13.00 | NA |  | 11.24 | NA |  | -1.76 (-2.51, -1.00) | NA | <.001 |

IPTW, inverse probability of treatment weighting; MIS, minimally invasive surgery; CI, confidence interval; OR, odds ratio; LOS, length of stay, ER, emergent room.
